# Morphology Changes During Sonication of Carbon Nanotube Suspensions and Impact on Heat Transport

**DOI:** 10.3390/nano16211425

**Published:** 2026-11-05

**Authors:** Panagiota V. Polydoropoulou, Vasilis N. Burganos

**Affiliations:** Foundation for Research and Technology, Hellas/Institute of Chemical Engineering Sciences (FORTH/ICE-HT), 26504 Patras, Greece; vbur@iceht.forth.gr

**Keywords:** carbon nanotube, sonication, numerical model, thermal management, deagglomeration, nanotube curvature, nanotube scission

## Abstract

Sonication of carbon nanotube (CNT) dispersions is commonly employed in practice to disentangle agglomerates in order to promote homogeneity and finer dispersion in suspensions and, consequently, to effect more predictable end-properties of nanofluids and nanocomposites. Sonication also influences CNT morphology in terms of shape and length, affecting, in turn, the thermal performance of the resulting medium. Although sonication is generally known to improve heat transfer, increased sonication times may have adverse results due to CNT buckling and scission and, thus, shortening of the CNTs. The present work aims to quantify the distinct effects of sonication-induced structural changes on the thermal conductivity and the evolution of this relationship in time through a dual-scale simulation approach. The simulator reconstructs three-dimensional (3D) assemblages of CNTs with prescribed agglomeration density, CNT length and curvature, evaluates the thermal conductivity locally and temporally within a representative volume element (RVE), and integrates the results into a macroscopic model to extract the effective conductivity as a function of sonication time. The numerical simulations are compared with experiments that follow the thermal conductivity as a function of sonication time. The results elucidate the separate but also the synergistic influence of the agglomerate microstructure on the effective thermal conductivity, paying particular attention to CNT curvature, length and agglomeration features. It is found that the internal density of the agglomerates is of major significance for the heat conduction process, followed by CNT length and curvature.

## 1. Introduction

Increased demands of modern applications include, most notably, advanced thermal management solutions, far beyond the conventional heating and cooling methods that are traditionally employed in a multitude of technological sectors [1]. Carbon-based nanofluids and nanocomposites represent promising alternatives in this direction thanks to their strong potential to exhibit exceptional thermal properties in practice [2]. In particular, carbon nanotubes (CNTs) are already considered in a wide range of applications for improved thermal management and in highly demanding environments [1], e.g., aerospace and automotive applications, high-performance electronics and electronic devices, thermal interfaces, energy storage, wearables, biomedicine, etc. As a result of their high aspect ratio and extremely high thermal conductivity, CNT-containing media can be architected and tailored to offer controlled levels of heat conduction or thermal insulation, depending on the application. Yet, during the preparation and processing of CNT-based suspensions, CNT properties can be degraded. Microscopy analysis after CNT processing has shown significant morphology changes, including buckling and scission and, therefore, shortening of the CNTs, structural damage of the first layers of CNTs [3], and organization of CNTs into different scales of agglomerates. Sonication, therefore, can lead to beneficial effects regarding the quality of the dispersion but also to morphological changes that may deteriorate the thermal performance [4,5]. As a consequence, the performance of CNT-based materials is often lower than expected. It is worth noting that following seemingly similar procedures, literature results have shown essential discrepancies in the end properties during operation, which necessitate the establishment of detailed protocols for the CNT processing [5,6,7].

Sonication uses ultrasound to disentangle CNT agglomerations within the suspension [6,8,9]. It is widely employed in the preparation of both nanofluids and nanocomposites, and more specifically in the incorporation of nanofillers into a solvent or carrier fluid, e.g., liquid resin, before further processing. Shear forces are applied to the fillers as a result of cavitation [10,11]. Small bubbles or voids are ruptured during sonication, leading to the gradual formation of small cavities. In addition to deagglomeration and disentanglement, these cavities can cause drastic scission of the CNTs and, hence, significant shortening. Among the key parameters governing the sonication process are the applied power and treatment time, as they affect energy input and energy density delivered during sonication [7]. The present work investigates how sonication time and the associated processing conditions influence the evolution of key CNT morphological features and, in turn, affect the thermal conductivity of the suspension.

The changes in certain morphological features of CNTs during the sonication process have been extensively investigated experimentally. For instance, it has been reported that increasing sonication time may break agglomerations or disentangle CNTs and lead to a more uniform dispersion [6,9,12]. However, at higher sonication times, CNT surface damage and shortened lengths have also been reported [3,4,10,11]. Furthermore, CNT curvature is also affected by sonication and appears to relax as sonication progresses. Curvature of long nanotubes plays an essential role in the effective distance that phonons can travel undisturbed, preferably along straight segments of CNTs. The precise sonication time for the resulting trade-off is far from established, owing to, among others, the lack of a standardized, optimal protocol for sonication treatments. For instance, Jozwiak et al. [13] found that the deterioration effect of the sonication time occurred after almost 3 min. Said et al. [14] showed that the thermal conductivity of functionalized MWCNTs-nanofluids depends non-monotonically on sonication duration, demonstrating an optimum sonication time depending on CNT concentration and temperature. Li et al. [15] also reported the existence of an optimum value during sonication time, after which further sonication has detrimental effects on the thermal behavior of nanofluids. Furthermore, Asadi et al. [16,17] studied experimentally the effect of prolonged sonication on thermal conductivity and stability of MWCNT–water nanofluids experimentally and found an optimum value of the sonication time at which the conductivity attains a maximum.

A significant parameter that requires further investigation is the internal, local volume fraction, *φ_local_*, of the CNT agglomerates, which is different from the macroscopic volume fraction, *φ_global_*, and from the volume fraction of agglomerates in the medium, *φ_bulk_* [18,19,20]. These three types of volume fraction are expected to affect the effective conductivity strongly and are related to each other through the following equation:

(1) $$\varphi_{bulk}=\frac{\varphi_{global}}{\varphi_{local}}$$

The majority of experimental studies [12,13,14,21,22,23] that investigated the effect of sonication on CNT-based materials report the overall resulting thermal behavior without making distinct reference to the contribution of the major individual mechanisms or parameters. This is evident from the nature of these factors, notably deagglomeration phenomena, changes in CNT curvature, and CNT shortening, and their simultaneous occurrence, in addition to local and bulk volume fractions of CNTs and agglomerates. More recently, Akbar et al. [22] investigated the effect of ultrasonication on MWCNT morphology, resulting in progressive nanotube shortening with increasing treatment duration. The resulting shortened CNTs exhibited improved dispersibility, highlighting the role of sonication-induced scission in modifying CNT morphology and dispersion behavior. Kim et al. [23] experimentally investigated the effect of ultrasonic frequency on the dispersion of SWCNTs in aqueous suspensions, reporting improved dispersion stability at 270 kHz compared with 400 and 700 kHz. Their findings highlight the influence of sonication conditions on CNT dispersion and the resulting properties. This is exactly where guidance by a numerical simulator can be pursued, as described in the present work. Although multiscale models have been used to investigate CNT agglomeration in CNT–polymer composites [19,24], to the authors’ knowledge, there is a dearth of studies on the effects of sonication-induced changes in CNT curvature, length, and agglomerate structure in three-dimensional RVEs, much less upscaled to predict the effective thermal conductivity of CNT suspensions as a function of treatment time. Other recent numerical studies have also investigated the thermal behavior of hybrid nanofluids using effective thermal conductivity models. Yasir et al. [25] studied the heat transfer characteristics of SWCNT–CuO/ethylene glycol hybrid nanofluids, whereas Sarfraz et al. [26] compared the predictions of the Xue and Hamilton–Crosser thermal conductivity models for hybrid nanofluids. These approaches describe the nanoparticle contribution through effective-property models, while the present study explicitly reconstructs CNT geometries within 3D RVEs to investigate the effects of CNT morphology and agglomeration on thermal conductivity.

The development of the present multi-scale numerical simulator is motivated by the difficulty in experimentally isolating the individual effects of the morphological changes occurring during sonication and their impact on the thermal behavior of the suspension. Although experimental measurements provide valuable information on the overall thermal performance of the suspension, it is difficult to determine separately the contributions of CNT curvature, shortening, and changes in the agglomerate structure. To this end, the proposed numerical model allows the investigation of these morphological characteristics independently at the agglomerate scale, while the resulting effective thermal properties are subsequently transferred to the next hierarchical scale, namely, the suspension scale. In this context, the model aims to clarify how the changes in CNT morphology during sonication affect the effective thermal conductivity of the suspension. A numerical simulator is developed here and used to investigate the separate effects of sonication-induced deagglomeration, CNT curvature changes, and CNT scission on the thermal conductivity of the medium. At the agglomerate scale, the temporal evolution of CNT curvature and shape, as well as that of the internal CNT concentration, is followed, and their effects on the thermal conductivity are evaluated. At the macroscopic level, the agglomerate properties are transferred to the nanofluid considering a stochastic distribution of agglomerates in the fluid with a volume fraction that is forced to satisfy the overall CNT mass balance, expressed in Equation (1). The numerical results at the suspension scale are compared with experimental data for the thermal conductivity as a function of sonication time, aiming (i) to assess the effectiveness of the numerical simulator to predict the thermal response of the full-scale suspensions, (ii) to highlight the effect of the internal agglomerate density on the overall thermal response and (iii) to show the extent to which the CNT curvature affects the macroscopic thermal behavior. For this purpose, numerical simulations in fully three-dimensional representative volume elements (RVEs) are performed, followed by similar computations at the suspension scale. One of the main contributions of this work is to reveal how the microstructural changes are related to the thermal performance of the whole suspension.

## 2. Methods

### 2.1. Overall Methodology

A dual-scale approach is developed to predict how the structural changes caused by the sonication process affect the effective thermal conductivity of CNT suspensions, considering the distinct and synergistic effects of improved dispersion and CNT straightening, but also of CNT scission and shortening. At the microscale, 3D representative volume elements are reconstructed and used for the computation of the effective thermal conductivity of CNT agglomerates. The agglomerate-scale approach builds upon previous algorithms that were developed for spherical nanoparticles and accounted for the effects of surfactants, dispersion and particle connectivity [27,28,29]. The results of this scale are integrated into a macroscopic model for the prediction of the overall performance of the CNT suspension, assuming a random dispersion of the CNT agglomerates in the base fluid. A schematic overview of the dual-scale approach is given in Figure 1.

As sonication time cannot be directly associated with heat transfer equations, specific morphology features of CNTs have been associated with sonication time, namely, the agglomerate size, the length of the CNTs, and the CNT curvature. The CNT diameter and length, as well as the agglomerate size at several sonication times, can be extracted from experimental results, e.g., [12]. On the contrary, CNT curvature and local CNT concentration within the agglomerates are harder to infer and are treated as parameters in the present work. Nevertheless, estimates are provided for the expected physical evolution of curvature and local volume fraction during sonication based on images and other literature information [12,30,31,32]. At early sonication stages, CNTs form large agglomerates and feature high curvatures as they keep, practically, their nominal length. As sonication proceeds, some CNTs are detached from the agglomerates and relax their curvature. At prolonged sonication, CNT scission and shortening can also become significant [3,5,10,11]. A commonly noticeable change is the buckling and scission of a large portion of CNTs, and the creation of shorter nanotubes. In fact, a semi-empirical expression has been suggested in the literature to relate CNT length reduction with sonication time that reads as follows [5]

*l(t) ~ t^−m^* (2)

where *m* is a parameter with values ranging around 0.3.

The internal density within the agglomerates (expressed as local volume fraction here, *φ_local_*) is relatively high in the beginning and reduces with time while peripheral CNTs, but also larger portions of the assemblages, start detaching gradually. Although some of these phenomena (such as CNT straightening and disentanglement) appear beneficial to the effective thermal conductivity, others (such as CNT shortening) are expected to hold back the conductivity enhancement. The methodology of the present work is deployed in a manner that allows distinct evaluation of these factors, aiming to elucidate their role and contribute to the design of tailored nanofluids and nanocomposites.

To facilitate the analysis and the extraction of practical conclusions from the simulator, the temporal evolution of a minimal set of parameters is used as input to the simulator, which eventually constructs the time profile of the thermal conductivity as sonication progresses. The temporal evolution of the effective conductivity is, subsequently, compared with the experiment. Specifically, data for the time dependence of CNT length and agglomerate size can be taken directly from the experiment, while CNT curvature and internal density of the agglomerates are left to be treated as parameters.

### 2.2. Agglomerate-Scale Model

CNTs are represented as arcs of axial symmetry, characterized by their curvature angle, *θ(t)*, radius, *R(t)*, and length, *l(t)*, as depicted in Figure 2. In addition to reducing the end-to-end length, CNT curvature can modify CNT-CNT thermal resistance; recent in-situ measurements demonstrate a strong relationship between CNT-CNT thermal resistance and overlap and contact morphology [33]. To simplify the analysis and be able to focus on the morphological features that are of interest here, the CNT-CNT contact resistance is neglected and isotropic conduction is assumed within the CNT bodies. Simulations at the agglomerate scale are implemented in a representative unit element (RVE) of orthogonal shape that contains a population of CNTs, which is statistically adequate for microscale computations of the local thermal conductivity. In the present work, populations of typically 80–430 CNTs proved to be sufficient depending on the volume fraction. Larger populations wouldn’t affect the results essentially, while, at the same time, they would require excessive amounts of computer memory and execution time, mainly due to the large aspect ratio of the CNTs (diameter in the range of 10–25 nm, length in the range of 250–2150 nm [12]).

For the generation of the CNT configurations at this scale, the candidate CNTs for insertion into the RVE are first assigned the desired geometrical features, namely, diameter, length, and curvature. They are subsequently inserted into the RVE on a sequential basis, at random positions and with random orientation, checking each time whether any overlap with previously deposited CNTs is detected. Periodic conditions for the placement of the CNTs are employed at all faces of the RVE. In addition to the usual merits of the periodic conditions that essentially extend the RVE results to much larger domains, the periodicity in the present case also facilitates the packing process, especially for relatively long CNTs compared with the RVE size. If overlap is detected, the insertion is canceled and a new set of position and orientation parameters is selected randomly. The insertion process is completed upon reaching the desired volume fraction, *φ_local_*, of the agglomerate. The in-house algorithm that was developed uses COMSOL Multiphysics 6.0 for the construction of the RVEs in line with the details that are discussed below and are given in a previous publication by the authors [20].

A typical RVE can be seen in Figure 3. The external radius of the torus is associated with the angle and curvature according to Equation (3),

(3) $$R(t)=\frac{180l(t)}{\pi \theta (t)}$$

where *R(t)* is the external radius of the toroidal geometry, *l(t)* is the length of the CNT and *θ(t)* is the angle of the torus (in degrees). A larger angle, *θ*, corresponds to more strongly curved CNTs, following the schematic of Figure 2. For *θ* = 0°, the CNTs become straight cylinders, whereas for *θ* = 360°, they form a closed circular torus.

At the agglomerate scale, the effective thermal conductivity is calculated by solving the steady-state heat conduction equation within the three-dimensional RVE containing CNTs and the surrounding base fluid, using the intrinsic thermal conductivities of the two phases. The effective conductivity is calculated from the temperature difference and heat-flux fields. The effective conductivity at the agglomerate scale is subsequently used as an input parameter in the suspension-scale simulations.

At each sonication stage, the following steady-state equation is solved numerically in 3D [34],

(4) $$\nabla \cdot \left(ki\nabla Ti\right)=0$$

where *k*_1_ and *T*_1_ are the conductivity and temperature in the fluid domain, and *k*_2_ and *T*_2_ refer to the CNT bodies, respectively.

For constant conductivity within each phase, in Cartesian coordinates, Equation (4) becomes [34]

(5) $$\frac{\partial^{2}Ti}{\partial x^{2}}+\frac{\partial^{2}Ti}{\partial y^{2}}+\frac{\partial^{2}Ti}{\partial z^{2}}=0$$

Periodic boundary conditions for the temperature and the heat flux have been imposed on all sides of the RVE except for the inlet and outlet faces where fixed temperatures are assigned (*T_in_, T_out_*) to drive heat flow. A temperature difference Δ*T* is applied between the two opposite faces of the RVE along the *x*, *y*, and *z* directions. The resulting heat flux is averaged over the RVE volume, and the directional effective thermal conductivity is calculated from the ratio of the corresponding averaged heat-flux component to the imposed macroscopic temperature difference, according to Fourier’s law. Three separate simulations are performed, with the temperature difference applied along the *x, y*, and *z* directions, to predict the directional effective thermal conductivities of each RVE.

Once the temperature profile is obtained numerically from Equation (5), the effective thermal conductivity in each direction is calculated from the equation

q = −k_x,y,z,e_ ∇T (6)

The total heat transfer rate $\dot{Q}$ is then calculated by integrating the heat flux over any face normal to the macroscopic gradient, say *A_inlet_*, according to Equation (7)

(7) $$\dot{Q}x=\int_{A}q_{x}dA$$

for the *x*-direction.

From Equations (6) and (7), the effective thermal conductivity becomes

(8) $$k_{x,e\;}=\frac{1}{A_{inlet}\Delta T}\left|\int q_{x}dA\right|$$

The overall effective conductivity of the RVE is obtained from orientational averaging, using

*k_eff_* = 1/3(*k_x,e_ + k_y,e_ + k_z,e_*) (9)

Local CNT volume fractions (*φ_local_*) of up to 4.5% *v*/*v* have been investigated, in line with volume fractions that are used in practice and also during experimentation. For each value, five random realizations are constructed and the thermal conductivity results are averaged to limit statistical deviation.

A Finite Element Method (FEM) has been employed to solve the steady-state Fourier heat conduction equation in the form of Equation (5).

### 2.3. Macroscale Level

The effective thermal properties that are calculated at the agglomerate scale are used as input to the macroscale simulator so that the effective conductivity of the suspension is obtained. The agglomerates are represented at this level as spherical inclusions. Other shapes can certainly be assumed, regular or stochastic, but the overall concept and approach remain the same. The mean effective thermal conductivity of five realizations that was calculated at the first scale for each set of parameter values is assigned as the thermal conductivity of the agglomerates within the medium at the macroscale. As was done at the RVE scale, five random realizations were also constructed at this scale, and their conductivities were averaged to yield the prediction of the overall effective conductivity. For the sake of comparison with experiments, the size of the aggregates at the macroscopic scale can be extracted from the SEM images at each sonication time, for instance, as done in [12]. It should be noted that as the local volume fraction of CNTs within the agglomerates increases, the agglomerate volume fraction within the medium is reduced and the CNT-free volume of the suspension increases according to Equation (1). As a result, a trade-off is expected, as will be discussed below. The numerical procedure for solving the heat transfer equations at this level, complemented by the same boundary conditions at the agglomerate/fluid interface and on the external faces of the working sample, is similar to the one that was used at the agglomerate scale.

### 2.4. Numerical Model and Validation

The numerical procedure is performed successively at the agglomerate and suspension scales. The effective thermal conductivity calculated from five independently generated CNT configurations at the agglomerate scale is averaged and subsequently assigned to the agglomerate domains of the suspension-scale RVE, where the overall effective thermal conductivity is determined.

The numerical model has been developed with the help of COMSOL Multiphysics 6.0 using the Heat Transfer in Solids and Fluids interface. A tetrahedral mesh has been employed for the agglomerate-scale simulations, as shown in Figure 4. The selected mesh settings included maximum and minimum element sizes of about 30 and 0.3 nm, respectively, a maximum element growth rate of 1.3, a curvature factor of 0.2, and a narrow-region resolution of 1. A mesh-sensitivity analysis was conducted using a representative RVE with a local CNT volume fraction of 1%. For this configuration, the selected mesh contained approximately 94 million elements with deviations less than 1% as compared with the refined mesh. The same element size parameters were applied to all configurations. Consequently, the total number of elements varied according to the CNT volume fraction and geometric complexity, increasing to approximately 350 million elements for RVEs with a local CNT volume fraction of 4%.

The simulation results for the evolution of the thermal conductivity with sonication time were compared with the experimental findings by Ruan and Jacobi [12]. The various parameters and the ranges that are used to this end are shown in Table 1.

For the characterization of CNT suspensions during sonication, electron microscopy images can be used to infer the average agglomerate size and CNT length at specific sonication times. These values can be used as input to the reconstruction stage of the simulator and serve to associate the evolution of the morphological features of the microstructure with sonication time. The curvature angle and the local CNT volume fraction are treated as parameters in the absence of direct experimental information. Different scenarios of the temporal evolution of these unknown parameters are studied, using, nonetheless, as a basic assumption that at early sonication stages the local volume fraction is relatively high and decreases with sonication time as CNTs and CNT bundles are disentangled. A similar change is assumed for the curvature angle, which is initially relatively high and relaxes with sonication. Indicative time profiles are shown in Table 1. The experimental mass fraction of 0.5 wt.% has been converted to volume fraction using typical densities of MWCNTs (2.1 g/cm^3^) and ethylene glycol (1.113 g/cm^3^). The thermal conductivity for the base fluid is set to 0.25 W/mK, which is a typical value for an ethylene glycol suspension, whereas for the CNTs, the thermal conductivity is set to 1000 W/mK [35,36]; both values are held constant during the simulations. The selected isotropic value represents an intermediate effective conductivity between the high longitudinal CNT conductivity, which can reach approximately 3000 W/mK, and the considerably lower radial conductivity.

Table 2 provides the values of $\varphi_{bulk}$ that are used at the macroscale, corresponding to the different values of the local volume fraction of the CNTs within the agglomerates through Equation (1). The total volume fraction, *φ_global_*, is kept constant during the simulations and equal to the value that is obtained from the experiments, as described above.

For the sake of direct comparison with experiments and in order to focus on the morphological parameters that are investigated here, the numerical and experimental thermal conductivities were normalized with respect to their corresponding values at a fixed sonication time, namely, 140 min. Consequently, the comparison is based on the relative evolution of the thermal conductivity during sonication, for both the simulations and the experiments.

Furthermore, a sensitivity analysis for the selection of the RVE size has been performed. The influence of domain size was examined using RVE side lengths ranging from 1250 to 1750 nm. A side length of 1500 nm was selected, as the calculated thermal conductivity differed by approximately 0.30% and 0.95% from the values obtained using side lengths of 1375 and 1625 nm, respectively. Figure 5 provides the results of the sensitivity analysis for the mesh and RVE selection.

More specifically, the numerical independence of the results was assessed through mesh-refinement and RVE-size sensitivity analyses, as presented in Figure 5. Five independently generated random CNT configurations were considered for each investigated combination of morphological parameters, and the corresponding effective thermal conductivities were averaged to reduce the influence of the random CNT realizations. With respect to the RVE side length, cubic domains with side lengths ranging from 1250 to 1750 nm were examined for CNTs of length 1000 nm to ensure adequate representation of the CNT geometries within the computational domain.

## 3. Results and Discussion

The efficiency of the numerical simulator to reproduce the evolution of the thermal conductivity of CNT suspensions during sonication was assessed first, based on experimental results [12]. The evolution of the effective thermal conductivity with sonication time is dictated by the synergy of the individual CNT morphology and the agglomerate microstructure, and their progressive change with time. The experimentally reported CNT lengths and agglomerate sizes were used to construct the RVEs at specific sonication times. The diameter of the CNTs was taken equal to the mean experimental value and set to 20 nm. In the case of curved CNTs, toroidal geometry with curvature angle ranging from 330° (almost closed torus) to 90° (highly relaxed torus) was considered. The selected conditions represent the expected physical evolution of the CNT suspension: as sonication progresses, the agglomerates become smaller and less dense, characterized by a decrease in the local CNT volume fraction. Simultaneously, the CNTs tend to become less curved and shortened, owing to the scission action of sonication and the concomitant agglomerate breakup. The numerical simulator combines these sonication-induced microstructural changes aiming to understand their effect at the suspension scale and predict the thermal conductivity at selected sonication times.

Figure 6 depicts the comparison of the simulation results with the experimental data extracted from the published results of Ruan and Jacobi [12], who reported that each thermal conductivity measurement was based on the average of five readings, with a two-standard-deviation (2σ) precision limit of ±3%. The comparison was performed at three sonication times corresponding to the experimental data. Three alternative time profiles for the local agglomerate volume fraction and the CNT curvature are investigated, as presented in Table 1. Based on the effective conductivity evolution with sonication time, it appears that the choice of the first set of parameter values provides the closest agreement with the experimental evolution of the thermal conductivity, with deviations of approximately 0.5% and 0.15% at 520 and 1355 min, respectively. This case corresponds to a progressive decrease in the local CNT volume fraction from 4.5% to 2.5% for sonication times of 140 min and 1355 min, respectively. Furthermore, highly curved CNTs (330°) tend to straighten gradually at high sonication times, reaching curvatures up to 90. This agreement indicates that the proposed combinations of agglomerate size, local CNT volume fraction, CNT curvature, and CNT length can efficiently represent the evolution of the normalized thermal conductivity. However, it is also evident that the results are very sensitive to changes of the CNT curvature and local volume fraction, as highlighted in Figure 6. Direct comparison of (a) and (b) shows that, for the same curvature profile, decreasing the local density within the agglomerates (tighter assemblages) would result in increased effective conductivity at the macroscale and increased deviation from experiment. On the other hand, direct comparison of (b) and (c) shows that increased relaxation of CNTs would cause a significant increase in effective conductivity and further deviation from experiment. These findings reveal that the effective thermal conductivity evolution during sonication proceeds as the result of the balance between the agglomerate microstructure, CNT curvature and CNT shortening.

Further to the experimental validation, the verified numerical simulator is used to study separately the effects of CNT curvature, length and local volume fraction, features that cannot be isolated directly from the experimental measurements, as it is difficult to conduct such physical testing. Yet, they are held responsible for the eventual thermal response at the macroscale. Therefore, the following parametric analysis aims to clarify the individual contribution of each morphological characteristic to the effective thermal conductivity.

### 3.1. Effect of CNT Curvature on Agglomerate Conductivity

During sonication evolution, entangled CNTs tend to disentangle and reduce their curvature. As shown in Figure 7, the effective thermal conductivity decreases monotonically with increasing CNT curvature angle. Larger curvature angles represent more strongly curved CNTs, with an angle of 360° corresponding to a complete circular geometry. Although the CNT length and aspect ratio remain constant, increased curvature represents a reduced effective end-to-end distance of the CNTs and, thus, results in less efficient conduction pathways through the agglomerate, in accord with previous studies demonstrating the adverse effect of CNT bending and buckling on thermal transport [37]. On the contrary, CNT straightening during sonication can improve thermal transport by providing longer and more direct conductive pathways.

### 3.2. Effect of CNT Length on Agglomerate Conductivity

The effective thermal conductivity of the CNT agglomerate increases with CNT length for all the curvature angles that were investigated in this work. Figure 8 shows the dependence of the conductivity on CNT length for two curvature angles. Longer CNTs provide extended heat conduction pathways through the agglomerate, thus enhancing thermal transport through CNT networking [38,39]. The results also depict the combined influence of length and curvature and suggest that longer and “straighter” CNTs within the agglomerates demonstrate the highest thermal conductivity. These findings indicate that CNT shortening due to scission during prolonged sonication adversely affects the thermal conductivity, partially counteracting the beneficial effects associated with CNT straightening and agglomerate breakup.

### 3.3. Effect of Local CNT Volume Fraction on Agglomerate and Macroscale Conductivity

At the agglomerate scale, increasing the local CNT volume fraction leads to a considerable increase in the effective thermal conductivity, owing to the greater number of highly conductive CNT pathways within the agglomerate and increased network connectivity [20,38] (Figure 9). However, for the same suspension, that is, for the same total CNT volume fraction, increasing the local CNT volume fraction corresponds to reduced bulk volume fraction of agglomerates in the medium and, consequently, to larger CNT-free regions within the base fluid. Therefore, the improvement observed at the agglomerate scale does not necessarily result in a similar increase in the effective thermal conductivity for the whole suspension. The results also indicate that the effect of relaxed CNT curvature becomes more pronounced at higher local volume fractions. This is due to the increased populations of less curved CNTs that offer a greater conductivity enhancement via the formation of more efficient heat conduction pathways.

The effect of the local CNT volume fraction within the agglomerates (*φ_local_*) on the effective thermal conductivity at the macroscale level is shown in Figure 10. The overall volume fraction of the nanofluid is kept constant so that one can isolate the effect of the local density on the overall thermal conductivity following upscaling from the agglomerate level. Following Equation (1), a specific value of the local CNT volume fraction corresponds to a specific volume fraction of the agglomerates in the suspension (*φ_bulk_*) for the same total density of the medium.

The bottom horizontal axis of Figure 10 refers to the local CNT volume fraction within the agglomerates (*φ_local_*), whereas the upper horizontal axis refers to the calculated agglomerate volume fraction (*φ_bulk_*). It is evident that increasing the local volume fraction produces more conductive individual agglomerates but, at the same time, reduces the volume of the suspension that is occupied by agglomerates. The result is the appearance of a trade-off that leads to maximum conductivity at *φ_local_* = 2%. It should be emphasized that the global CNT volume fraction remains constant at 0.266% throughout these simulations; therefore, the observed decrease in thermal conductivity beyond this maximum is associated with the increasing proportion of CNT-free base fluid rather than an increase in the total CNT loading. The immediate conclusion from this graph is that the thermal conductivity is maximized at intermediate conditions between fully dispersed CNTs and fully agglomerated CNTs, the precise state being certainly dependent on the rest material features, including overall volume fraction, CNT length and CNT shape. A similar observation was made in experimental studies [13,17], considering that the agglomerate density decreases as sonication progresses.

## 4. Conclusions

A dual-scale numerical framework was developed in the present work aiming to investigate the main morphological effects of sonication on CNT dispersions, and how these affect, in turn, the effective thermal conductivity of the nanofluid or nanocomposite, depending on the application. At the first scale, 3D RVEs containing randomly dispersed CNTs are constructed and the thermal conductivity of individual agglomerates is calculated by solving the heat conduction equation numerically, considering as main parameters the local CNT volume fraction, the CNT curvature, and the CNT length. At the macroscale, the calculated agglomerate properties served as input to the full-scale suspension simulator, that views the suspension as an assemblage of agglomerates and solves again the heat conduction equation. At this scale, the volume fraction of agglomerates in the medium is extracted from the relation between the local volume fraction and the global one.

The results showed that the CNT morphological features have a significant effect on heat transfer at both scales. Increased CNT curvature led to lower effective thermal conductivity, as strongly curved CNTs provide shorter and more curved pathways for heat transfer. Lengthier CNTs resulted in higher thermal conductivity by providing longer conductive pathways. Therefore, CNT straightening during sonication can have a beneficial effect on heat transfer, while CNT shortening as a result of scission can lead to the deterioration of the thermal performance of the whole suspension. Furthermore, increasing the local CNT volume fraction improved the thermal conductivity of the individual agglomerates due to the increased number of highly conductive CNT pathways.

Nevertheless, the macroscale results revealed that the increase in the local CNT volume fraction does not necessarily lead to an increase in the thermal conductivity of the whole suspension. At a constant total CNT volume fraction, increasing the local CNT volume fraction reduces the volume fraction of the agglomerates in the medium, thus increasing the low-conducting, CNT-free regions within the suspension. As a result, the effective thermal conductivity showed a non-monotonic behavior and is maximized at intermediate conditions between fully dispersed CNTs and fully agglomerated CNTs. Therefore, a fixed total CNT volume fraction cannot provide a specific thermal conductivity value, as the final performance also depends on the local volume fraction and microstructure of the CNTs within the agglomerates.

For the validation of the simulator, the numerical predictions were compared with available experimental measurements for MWCNT–ethylene glycol suspensions at different sonication times. The CNT length and agglomerate size that were experimentally measured at different sonication times were used as input to the simulator for the prediction of the suspension-scale thermal conductivity. Satisfactory correlation with experiment was achieved. Specifically, it was found that the deviation of the simulated, normalized thermal conductivity from the experimental values along the progress of sonication was of the order of 1% for selected values of the agglomerate density and CNT curvature. The results showed an increase in the suspension conductivity with sonication time, which was further enhanced during the sonication intervals investigated here. Yet, the progressively lower rate of the increase in the effective thermal conductivity at high sonication times indicates a balancing effect between deagglomeration and CNT scission that are caused by sonication.

The numerical simulator developed in this work enables the separate evaluation of the microstructural changes that take place during sonication and the concomitant changes of the thermal conductivity at the agglomerate and the suspension scale. The dual-scale approach allows the transfer of the agglomerate properties to the macroscale, establishing relationships between CNT morphology, agglomerate structure and the thermal performance of the whole suspension. The incorporation of experimentally determined CNT curvature and local CNT volume fraction in future studies could further extend the capability of the model to guide the optimization of sonication conditions for the preparation of nanofluids and nanocomposites.

## Figures and Tables

**Figure 1:**
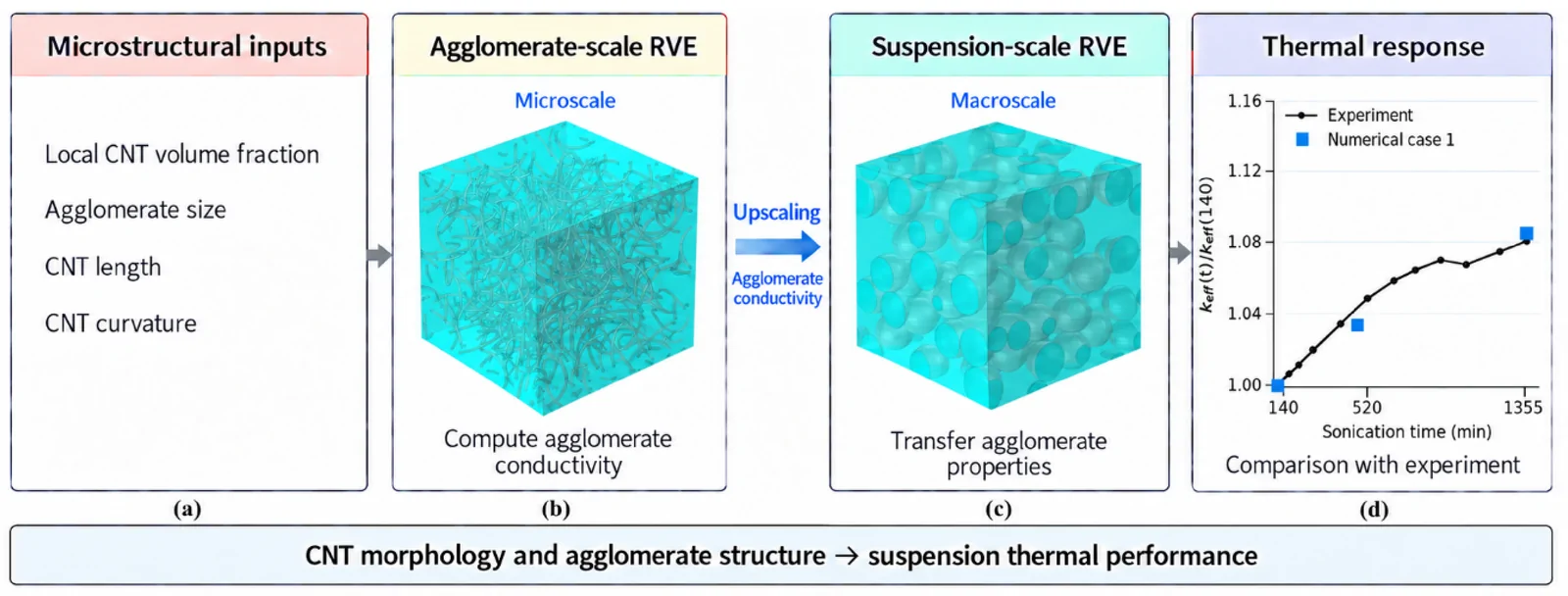
Schematic overview of the dual-scale simulator. (**a**) Microstructural features include CNT morphology and local concentration. (**b**) Effective conductivity is computed at the RVE level. (**c**) The macroscale corresponds to the spatial distribution of agglomerates and is used to obtain the thermal conductivity as a function of sonication time. (**d**) Comparison of the time evolution is made against experimental data.

**Figure 2:**
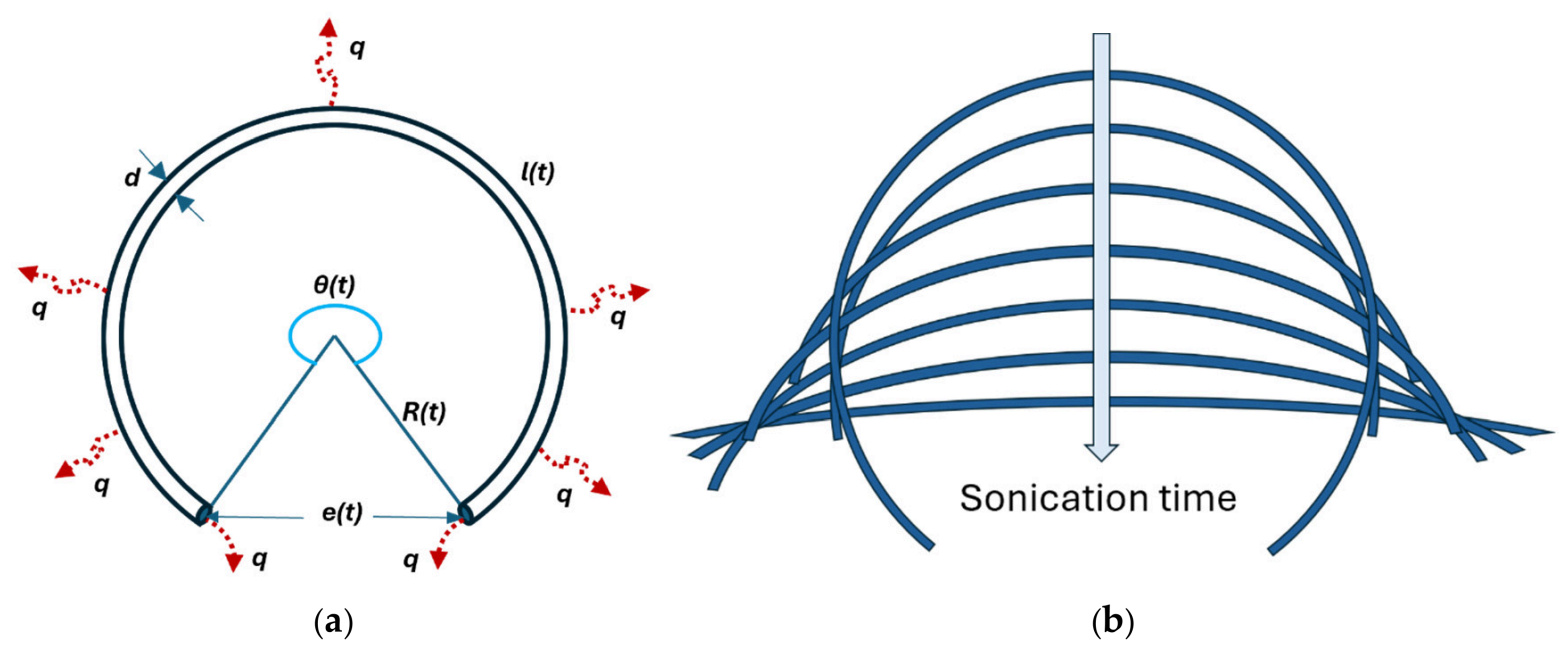
(**a**) Schematic representation of the assumed evolution of CNT curvature and length, showing the main geometrical features and heat fluxes. (**b**) Reduction of both curvature and length with sonication time.

**Figure 3:**
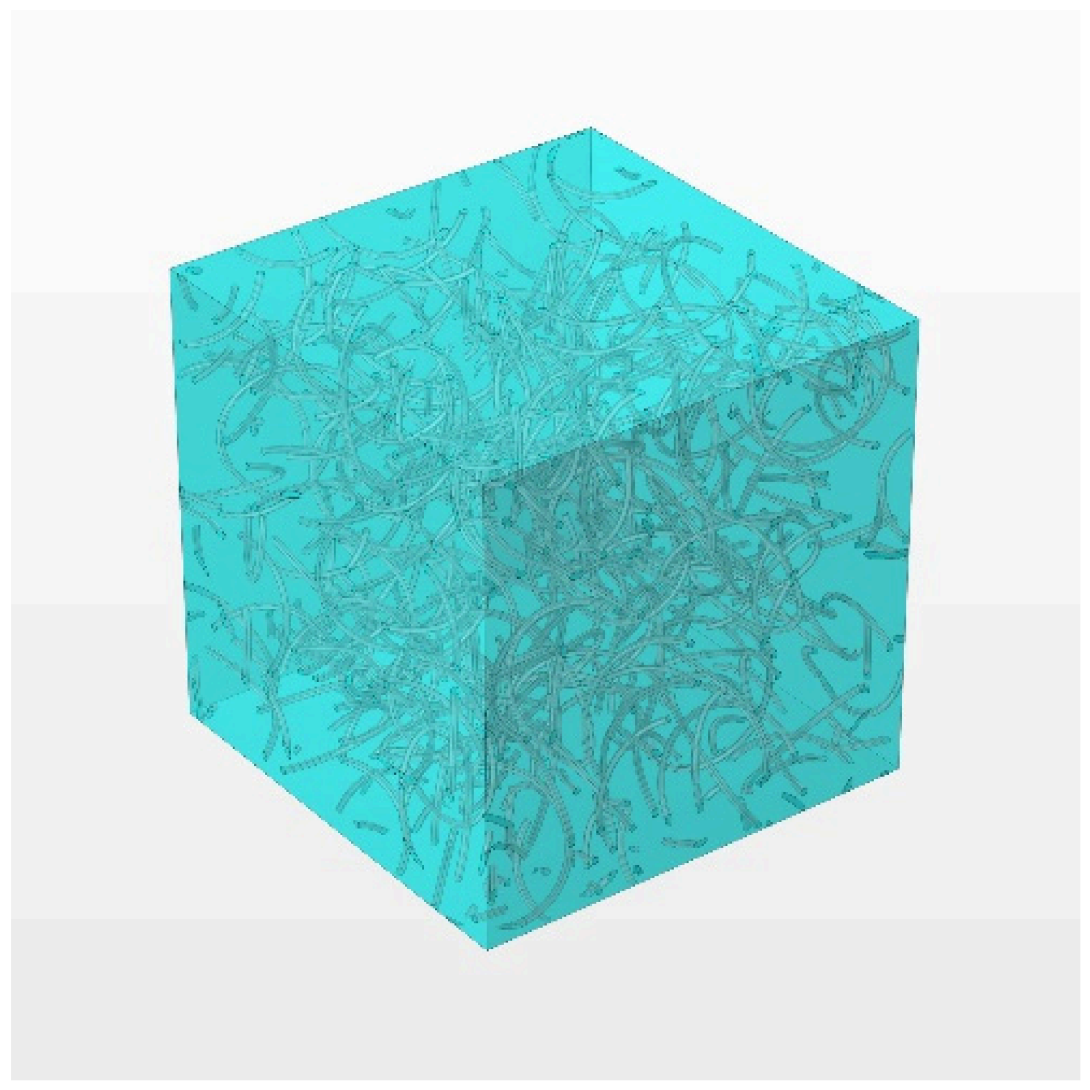
Representative 3D periodic RVE of a CNT agglomerate containing randomly oriented curved CNTs.

**Figure 4:**
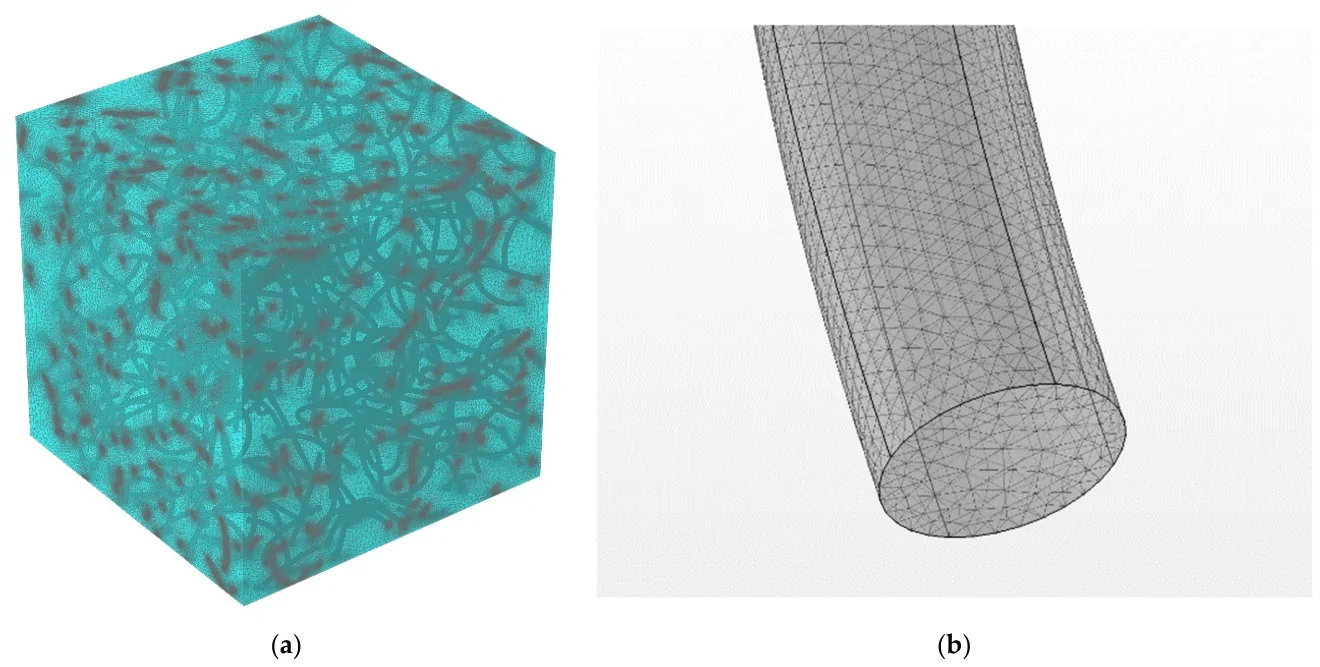
Representative finite element discretization of the agglomerate-scale model: (**a**) Typical mesh of an RVE containing randomly oriented CNTs and (**b**) local mesh refinement around a CNT.

**Figure 5:**
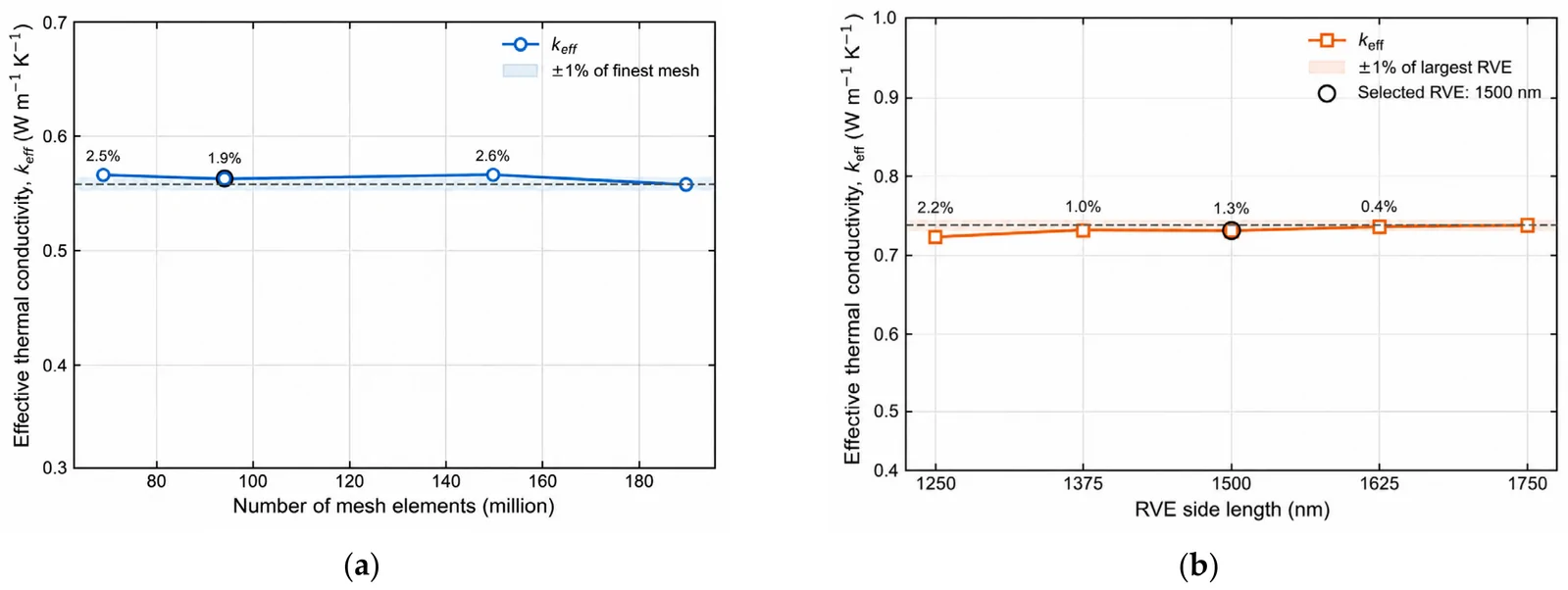
Numerical convergence of the agglomerate-scale model: (**a**) Mesh-sensitivity analysis for a local CNT volume fraction of 1%. (**b**) Variation of the effective thermal conductivity with the RVE side length.

**Figure 6:**
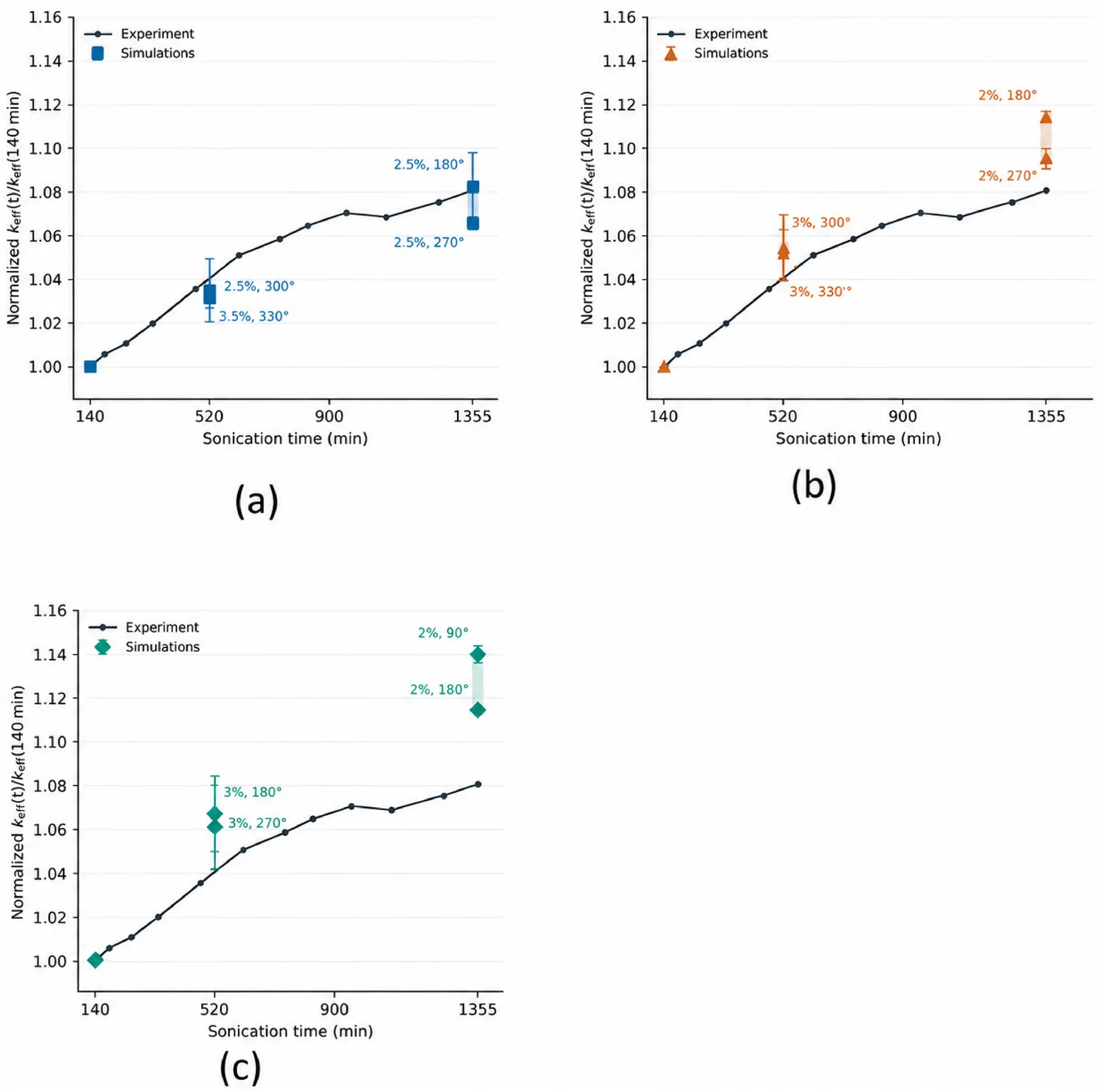
Comparison of the normalized macroscale effective thermal conductivity predicted for different time profiles from Table 1, with the experimental measurements in [12] during sonication. (**a**,**b**) isolate the influence of the local CNT volume fraction for constant CNT curvature angle; (**c**) isolates the influence of CNT curvature. Numerical and experimental conductivities are normalized by their respective values at 140 min.

**Figure 7:**
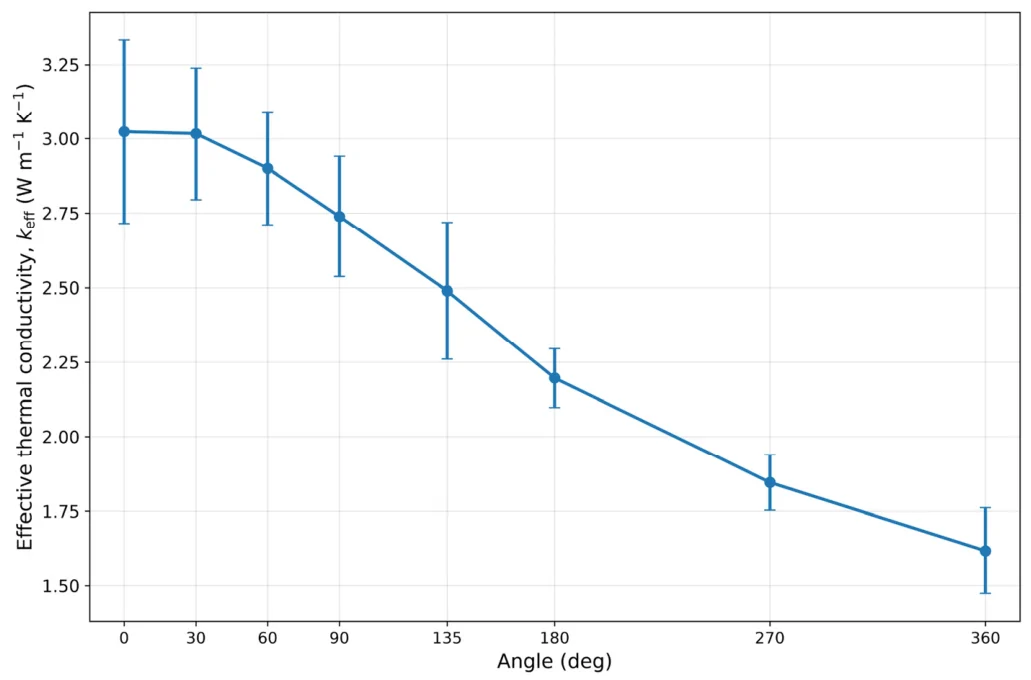
Agglomerate-scale effective thermal conductivity as a function of CNT curvature angle for a CNT length of 1000 nm and a local CNT volume fraction of 2%. An angle of 0° represents a straight CNT, while 360° represents a fully circular CNT geometry.

**Figure 8:**
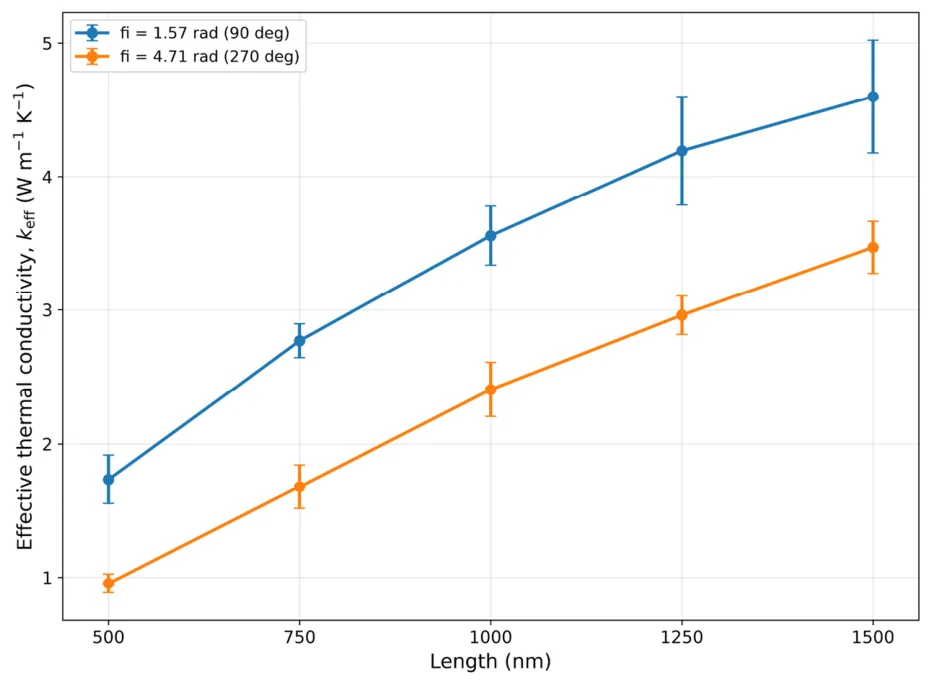
Agglomerate-scale effective thermal conductivity as a function of CNT length at a constant local CNT volume fraction of 2.5%. Results are presented for curvature angles of 1.57 rad (90°) and 4.71 rad (270°). Error bars represent the standard deviation of the numerical results for five realizations.

**Figure 9:**
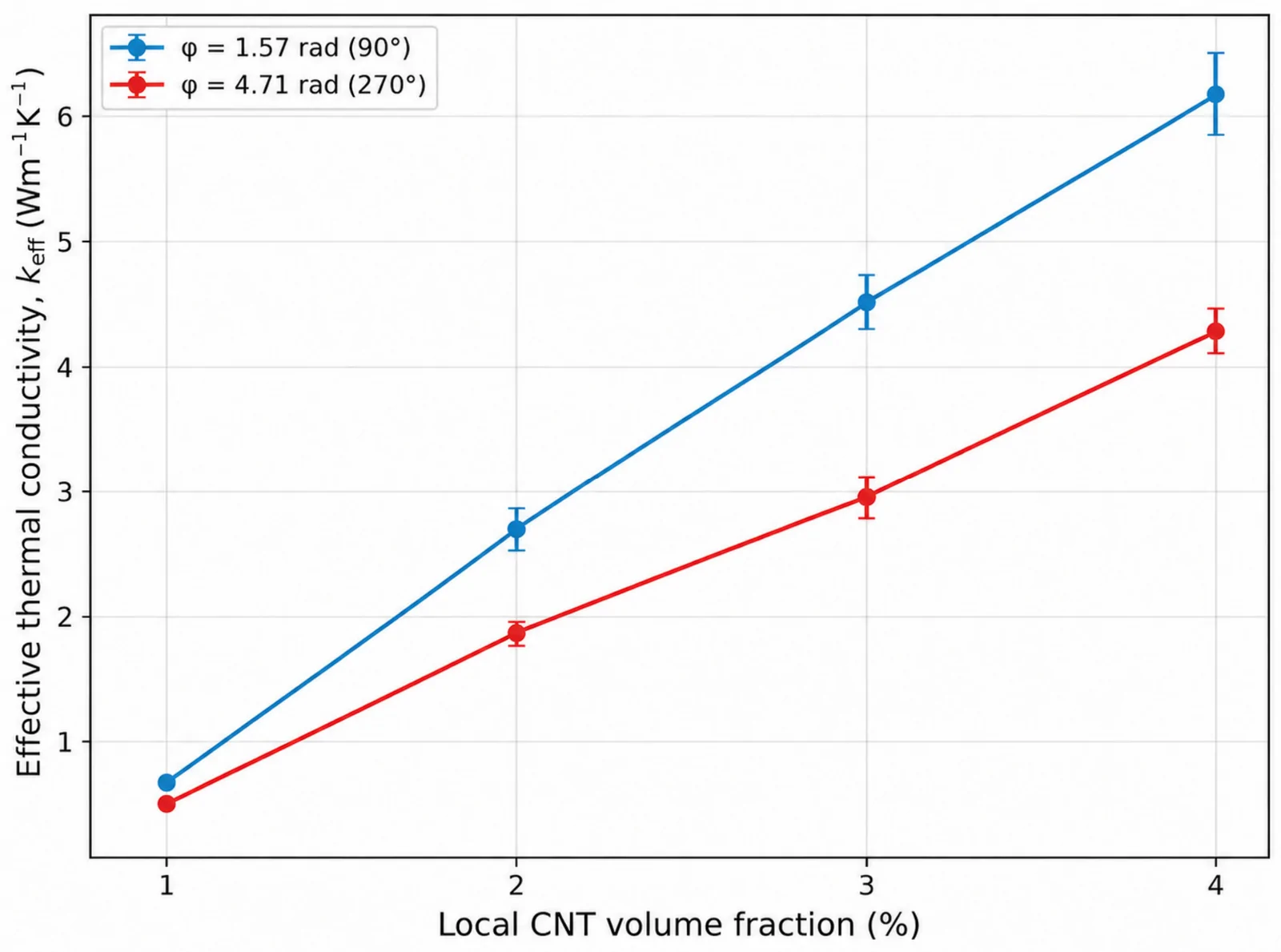
Agglomerate-scale effective thermal conductivity as a function of the local CNT volume fraction for curvature angles of 1.57 rad (90°) and 4.71 rad (270°). The CNT length was kept constant at 1000 nm.

**Figure 10:**
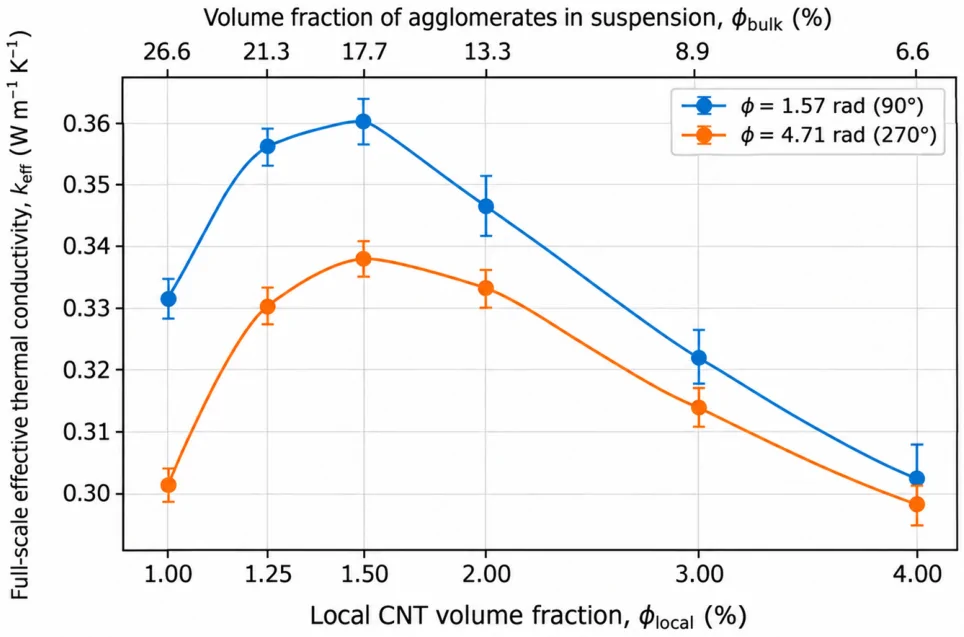
Dependence of the effective thermal conductivity at the suspension scale on the local CNT volume fraction within the agglomerates. The total CNT volume fraction in the suspension is kept constant at 0.266%. The volume fraction of agglomerates in the suspension is calculated from Equation (1). Variation with CNT curvature angle.

**Table 1:** CNT morphological and agglomerate parameters considered at different sonication times for the comparison with experimental data.

Time (min)	140	520	1355
CNT length [12] (*l*: nm)	2150	1500	1000
Local CNT fraction (*φ_local_*: %)	4.5 4.0	3.5 3.0	2.5 2.0
Curvature angle (*θ*: degrees)	340 330	330/300 270/180	270/180 180/90
Agglomerate radius [12] (*R_agg_*: nm)	3750	1500	1000

**Table 2:** Volume fraction of CNTs in the medium (total volume fraction, *φ_global_*), local volume fraction within the agglomerates (local volume fraction, *φ_local_*), and volume fraction occupied by the agglomerates in the medium (bulk volume fraction, *φ_bulk_*).

Total Volume Fraction of CNTs in Medium [12], *φ_global_*	Local Volume Fraction of CNTs in Agglomerates, *φ_local_*	Bulk Volume Fraction of Agglomerates in Medium, *φ_bulk_*
0.266%	1%	26.6%
0.266%	2%	13.3%
0.266%	3%	8.87%
0.266%	4%	6.65%

## Data Availability

The original contributions presented in this study are included in the article. Further inquiries can be directed to the corresponding author.
